# Using Augmented Reality Toward Improving Social Skills: Scoping Review

**DOI:** 10.2196/42117

**Published:** 2023-09-20

**Authors:** Gloria Mittmann, Vanessa Zehetner, Stefanie Hoehl, Beate Schrank, Adam Barnard, Kate Woodcock

**Affiliations:** 1 Die offene Tür Research Group for Mental Health of Children and Adolescents Ludwig Boltzmann Society at Karl Landsteiner University of Health Sciences Krems Austria; 2 Research Centre Transitional Psychiatry Karl Landsteiner University of Health Sciences Krems Austria; 3 Department of Developmental and Educational Psychology University of Vienna Vienna Austria; 4 Department of Psychiatry and Psychotherapeutic Medicine University Hospital Tulln Tulln Austria; 5 School of Psychology University of Birmingham Birmingham United Kingdom

**Keywords:** virtual reality, serious games, autism spectrum disorder, social learning, communication, cooperation, mobile phone

## Abstract

**Background:**

Augmented reality (AR) has emerged as a promising technology in educational settings owing to its engaging nature. However, apart from applications aimed at the autism spectrum disorder population, the potential of AR in social-emotional learning has received less attention.

**Objective:**

This scoping review aims to map the range of AR applications that improve social skills and map the characteristics of such applications.

**Methods:**

In total, 2 independent researchers screened 2748 records derived from 3 databases in December 2021—PubMed, IEEE Xplore, and ACM Guide to Computing Literature. In addition, the reference lists of all the included records and existing reviews were screened. Records that had developed a prototype with the main outcome of improving social skills were included in the scoping review. Included records were narratively described for their content regarding AR and social skills, their target populations, and their outcomes. Evaluation studies were assessed for methodological quality.

**Results:**

A total of 17 records met the inclusion criteria for this study. Overall, 10 records describe applications for children with autism, primarily teaching about reading emotions in facial expressions; 7 records describe applications for a general population, targeting both children and adults, with a diverse range of outcome goals. The methodological quality of evaluation studies was found to be weak.

**Conclusions:**

Most applications are designed to be used alone, although AR is well suited to facilitating real-world interactions during a digital experience, including interactions with other people. Therefore, future AR applications could endorse social skills in a general population in more complex group settings.

## Introduction

### Background

Digital technology has begun to move beyond the constraints of static, screen-based media toward more dynamic, immersive environments such as virtual reality (VR). One specific technology that is embedded in the concept of VR and is being increasingly used in various sectors is augmented reality (AR). AR creates visual situations where virtual components overlap with the real world, inviting the user’s complicity in the illusion that these virtual components exist in the real world. As such, AR does not create a virtual environment per se but acts as a digital supplement to the real environment [1], clearly distinguishing it from VR. Its accessibility makes it easier to use than, for example, VR while still immersing the user in a partly virtual world. A wide range of technology supports AR. Although in the early stages, AR was a form of VR with a head-mounted display [2], current advanced hardware and software deliver AR through handheld devices, including tablets and mobile phones [3]. AR can be either marker based (activated through a specific physical image pattern, such as a QR code) or markerless. Markerless AR uses location-based AR (eg, via GPS), projection-based AR (tied to a specific physical space), or superimposition AR (replacing an object with an augmented view, eg, a real face with a cartoon face).

These advances have made AR a successful technology for commercial gaming (the most popular being the 2016 Pokémon Go by Niantic) and for informal learning sites, such as exhibits or museums to increase interaction with showcased topics [4]. In addition, the use of AR in formal educational settings such as classrooms has increased because of the cost-effective, interactive, and innovative features of AR (for literature reviews, refer to studies by Koutromanos et al [5] and Laine [6]). In their review of AR and education, Garzón et al [7] found that most AR technology is used in the field of natural sciences, highlighting the focus of current educational AR applications.

### Social Skills

Social skills are crucial throughout life for social functioning [8] and for forming and maintaining relationships and reacting appropriately in social interactions through successful communication and socioemotional functions [9]. Furthermore, social skills relate to other important concepts: during childhood, they influence learning motivation and academic achievements and competences [10,11], whereas a lack of social skills can lead to mental health difficulties [12] such as depression [13]. Moreover, social competence in childhood is significantly related to adult well-being [14].

There is no commonly shared definition for social skills. Various terms are often used synonymously to refer to the concept of social skills, including *social competence*, *soft skills*, *emotional learning*, or *social behavior* [15], although some authors argue that these terms cannot be used interchangeably [16]. Most definitions of social skills include successful communication and adaptive interactions with others, and researchers agree that social skills are acquired through specific learned behaviors that are also socially reinforced [17]. For example, Bedell and Lennox [18] described social skills as “the ability to (a) accurately select relevant and useful information from an interpersonal context, (b) use that information to determine appropriate goal directed behavior, and (c) execute verbal and nonverbal behaviors that maximize the likelihood of goal attainment and the maintenance of good relations with others.” Grover et al [19] synthesized existing definitions into 4 core social skill areas for a general population: communication skills, emotion regulation, cognitive skills, and social problem solving. In the literature search for this review, we concentrated on the 2 terms *social* and *communication* as the vital parts of social skills [9,17].

### Social Skills and AR

Analog social and emotional skills training interventions have been successfully implemented, for example, in preschool children. The results of a meta-analysis showed small to medium effects of these interventions on social skills development and problem behavior reduction [20], demonstrating the potential of using interventions to improve social skills. Furthermore, serious games without an AR focus also cover so-called *21st-century skills* [21], such as media and technology skills or learning and innovation skills (eg, critical thinking and collaboration). Although social-emotional learning trainings are often developed for a nondigital classroom setting, AR could also be particularly interesting in the field of social-emotional learning. First, using a digital approach with AR allows a controlled setting while still only partly immersing the user in a digital environment, thus making the setting more realistic. Second, it harnesses people’s increasing interest in digital tools while still being able to engage them in real-world complex social interactions. A look at the positive effects of existing commercial AR games on sociability offers promising results; for example, Ewell et al [22] found in a 7-day diary study on the commercial AR game *Pokémon Go* that the time spent with the game was connected to more social interactions, and Ruiz-Ariza et al [23] found that *Pokémon Go* significantly increased sociability levels and good social relationships.

In the field of psychology, AR is primarily used as a successful form of exposure therapy for phobias such as fear of small animals, for example, cockroaches and spiders [24], or as an additional tool to treat posttraumatic stress disorder [25]. Little focus has been put on improving social or emotional skills. In terms of social-emotional learning, most existing literature related to AR focuses on improving social skills in populations diagnosed with autism spectrum disorder (ASD). In a systematic review of AR applications for children and adolescents with autism by Khowaja et al [26], 20 of 30 included studies reported outcomes related to social skills and competences. Considering the existing deficits in social skills in people with autism [27], it makes sense to develop and implement interventions specifically for this population. Yet, social skills are generally essential for all populations. Consequently, the question arises if and what AR applications that improve social skills are being developed without a specific focus on an autistic population.

### This Study

The challenges of identifying relevant literature due to the interdisciplinarity of the topic, the different denominations of social skills, and the existing focus on ASD in existing reviews highlight the need to map out the existing literature on AR and social skills. Thus, the aim of this scoping review was to systematically identify the range and characteristics of records that report the use of AR to improve social skills. Specifically, within this literature, our objective was to answer the following questions: what are the characteristics? What are the target groups? What types of AR are being used? What social skills are being targeted? What social settings are being targeted? What are the methodological characteristics of the studies evaluating the applications? We followed the guidelines for conducting systematic scoping reviews by Peters et al [28]. The PRISMA-ScR (Preferred Reporting Items for Systematic Reviews and Meta-Analyses extension for Scoping Reviews) checklist can be found in Multimedia Appendix 1.

## Methods

### Data Sources and Search Strategy

#### Initial Limited Search

The first step was to identify the strings and terms that identify most of the relevant literature to answer the research question. We used a nonsystematic literature search to come up with our search strings for social skills, “social” and “communication.” We further started manually with a few articles that we found through existing reviews and an initial search that would fit our inclusion criteria; looked at their keywords, references, and the terms they used; and collected the most used and most relevant terms.

#### Included Databases

We used 2 main databases for our search: one was intervention focused, that is, PubMed, and the other leaned toward computer science, that is, IEEE Xplore. After double-checking and realizing that some of our initial articles could not be found in either of these databases, we decided to also include a third database: the ACM Guide to Computing Literature. We selected these databases to comprehensively cover all relevant disciplines.

#### Primary Search

Our final search terms included “augmented reality” plus “social” or “communication” (derived from definitions of social skills) or “collaboration” or “cooperation” (derived from keywords related to AR). The decision to not use the word “skill” in the search was intentional to capture a wider range of relevant terms, such as “social competence.” We limited our search to the period from 2010 to December 2021. Owing to advancements in technology, AR has only become available for use on everyday smartphones during the last decades, and nowadays, applications are hardly comparable with the first versions of AR in the 1990s and 2000s. Specific search strings for each database are listed in Table 1. The search strategy was supervised by an experienced researcher (KW).

**Table 1:** Search strings used in the different databases.

Database	Search string	Filters
PubMed	(“augmented reality”) AND ((soci*) OR (collaborat*) OR (communicat*) OR (cooperat*))	≥2010
IEEE Xplore	(“Abstract”: “augmented reality”) AND ((“Abstract”:soci*) OR (“Abstract”:collaborat*) OR (“Abstract”:communicat*) OR (“Abstract”:cooperat*))	≥2010
ACM Guide to Computing Literature	[Abstract: “augmented reality”] AND [[Abstract: soci*] OR [Abstract: collaborat*] OR [Abstract: communicat*] OR [Abstract: cooperat*]] AND [Publication Date: (01/01/2010 TO *)]	—^a^

^a^Not available.

#### Additional Search

In addition to the primary search, we screened existing recent reviews on AR [7,26,29-41] as well as reference lists of all included papers for additional references and searched for follow-up papers of records that were excluded because they did not reach the development phase of the application.

### Citation Management

All references were imported and processed using EndNote20 (Clarivate) [42]. Duplicates were removed using the program’s function to find duplicates and by manually going through all references. In the first step of screening, 2 independent reviewers (GM and VZ) screened all references on a title, and in a second step, 3 independent reviewers (GM, VZ, and a group of 3 research group members) screened all references on an abstract level. In the third step, the reviewers screened the remaining references on a full-text level. Discrepancies between reviewers were resolved through discussions after each step.

### Eligibility Criteria

The eligibility criteria were developed before the screening process and applied to all stages of the review. Some modifications were made to the eligibility criteria during the screening process, as described in the *Results* section. This review included all records that met the inclusion criteria, as described in Textbox 1.

**Eligibility criteria.**

**Inclusion criteria**

- Studies related to the research questions, that is, described the use of augmented reality (AR) to improve social skills, defined as successful communication and adaptive interpersonal interactions
- The application has at least reached the end of prototype development
- Social skills were the goal of the application and the main outcome of the study (added after the first round of abstract screening)
- Records were in English, German, Portuguese, or Spanish

**Exclusion criterion (added after the first round of abstract screening)**

- The AR applications scaffold aspect of social functioning without actually improving a social skill in a transferable manner

### Data Characterization

For all included articles, the following study characteristics were retrieved: general characteristics (authors, title, year, country of origin, sector, and paper length); application information (type, name, description of application, description of AR part, development software, duration, description of social skill, description of social setting, description of other outcomes if any, and co-design if any); study information (population, setting, design, methods, analysis, results, and effect sizes if any); and challenges and limitations. The data were summarized in an Excel (Microsoft Corporation) spreadsheet. Statistical analysis was performed using SPSS Statistics 27 (IBM Corp).

## Results

### Search and Selection of Articles

The primary search was conducted on December 2, 2021, and yielded 2748 potential records. The main reason for exclusion at the abstract level was that the AR system did not address social skills as an outcome. For example, although many articles had keywords in their abstracts, “collaboration” was only used as a means to teach other outcomes, “cooperation” tackled human-machine interactions, or “communication” related to a technical term (eg, network communication). The low reviewer agreement after the first round of abstract screening (500 vs 69 included records after abstract screening) resulted in refining the eligibility criteria and repeating the abstract screening process. Specifically, the initial inclusion criteria included all records that had social skills as one outcome instead of the main outcome. This change was made because many included references had little to do with social skills (>900). Furthermore, an exclusion criterion was added to exclude AR applications that only supported existing communication by adding AR elements (such as adding an AR avatar to a web-based communication tool or including information about the current conversation partner, such as their hobbies), as we argue that these applications only support communication in that specific situation instead of improving social skills in a transferable manner.

**Figure 1:**
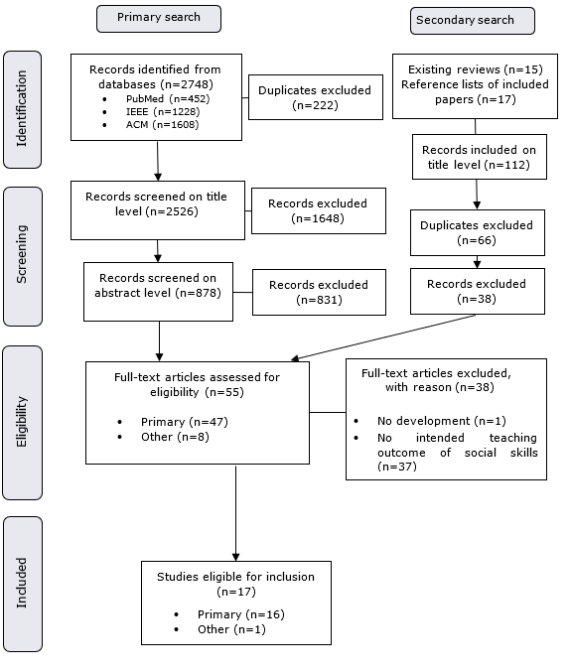
A flowchart of the record selection process.

After refinement of the eligibility criteria, in addition to the 2 main reviewers GM and VZ, a group of 3 research members collectively screened all abstracts and full texts to ensure the sufficiency of the new criteria. This was done to include naive reviewers, as the new eligibility criteria were developed by the first 2 reviewers who may have been more likely to agree. Decisions during the screening process were categorized as “yes (include),” “maybe,” or “no (exclude).” Cohen weighted κ was used to evaluate the reviewer agreement, and linear- and quadratic-weighted κ coefficients were reported to specify the shape of the disagreement distribution for the 3-ordinal scale (“yes,” “maybe,” and “no”) [43]. Table 2 presents the agreement between the 3 reviewers at the abstract and full-text levels. The agreements ranged from moderate (Cohen weighted κ=0.594) to almost perfect (Cohen weighted κ=0.861) [44]. After full-text screening, a total of 17 records were included in this review (for the selection process, see Figure 1).

**Table 2:** Reviewer agreement on the abstract (second round) and full-text levels.

	Abstract level (linear-weighted κ/quadratic-weighted κ)	Full-text level (linear-weighted κ/quadratic-weighted κ)
Reviewer 1 (GM)–Reviewer 2 (VZ)	0.594/0.689	0.766/0.842
Reviewer 1 (GM)–Reviewer 3 (RG^a^)	0.628/0.817	0.794/0.861
Reviewer 2 (VZ)–Reviewer 3 (RG)	0.775/0.640	0.792/0.820

^a^RG: research group members.

### General Characteristics of Included Records

The general characteristics of all included records are presented in Table 3. All included records were published between 2012 and 2021, although notably, only 2 included records were published before 2015, emphasizing the recent increase in the interest in AR. Most records have their origins in Asia and North America, and the majority have been published via conference proceedings (with journals primarily publishing with a focus on an autistic population), although they were relatively evenly spread between the social sciences, computer science, and interdisciplinary sectors.

**Table 3:** General characteristics of included records (N=17).

		Values, n (%)	Reference
**Year of publication**			
	2010-2014	2 (12)	[45,46]
	2015-2017	8 (47)	[47-54]
	2018-2021	7 (41)	[55-61]
**Origin of reference**			
	Asia	8 (47)	[45,48,52,53,55,57,60,61]
	Europe (including the United Kingdom)	3 (18)	[47,49,51]
	North America	5 (29)	[46,54,56,58,59]
	South America	1 (6)	[50]
**Type of publication**			
	Journal article	7 (41)	[48,50,52,54,59-61]
	Conference proceedings	9 (53)	[45-47,49,51,53,55,56,58]
	Other	1 (6)	Book chapter [57]
**Sector**			
	Social science	4 (24)	[48,54,59,61]
	Computer science	5 (29)	[50,51,53,55,56]
	Interdisciplinary	8 (47)	[45-47,49,52,57,58,60]

### Characteristics of AR Applications to Improve Social Skills in Autistic Populations

Of the 17 included records, 10 targeted autistic populations. As AR applications for this population have already been the subject of different reviews (although not specifically focused on social skills) and because of the unique social challenges faced by individuals with autism, we examined their characteristics separately to provide an overview of applications designed for autistic populations (Table 4). All records included in the autistic populations describe applications developed for children. Most of the applications (n=4) aimed to teach facial or emotional expressions as a social skill, often focusing on the basic emotions (happiness, sadness, fear, disgust, surprise, and anger). For instance, in *FaceMe* [55], children play minigames in which players must remember virtual expressions, identify expressions, choose the right expression, and imitate expressions. Similarly, in *Brain Power System (BPS)* [54], children learn to identify facial expressions in a gamified environment by selecting the right emotional expression for a virtualized human, and in *GameBook* [51], the emotional expressions are picked for a fictional character. In *augmented reality-based self-facial modeling* [48], the participants see their own virtual faces that can be overlaid with AR facial expressions in fictional scenarios, and in *augmented reality-based video modeling storybook* [52], social cues are taught by using a storybook that can be augmented with additional AR video clips.

**Table 4:** Characteristics of augmented reality application for an autistic population (n=10).

Study, year	Name	Type	Targeted social skill	Description of application	Target group (all individuals with autism)
Chen et al [48], 2015	ARSFM^a^	Mask or self-facial modeling	Facial or emotional expressions	Users choose facial masks that fit to emotions of a short story and see their faces overlaid with the emotion	Age 10-13 y
Chen et al [52], 2016	ARVMS^b^	Virtual book	Facial or emotional expressions	Users train to recognize significant social cues in a digital storybook and short video stories	Age 11-13 y
Cunha et al [51], 2016	GameBook	Virtual book	Facial or emotional expressions	The user learns to identify and choose emotions of a fictional character in different parts of the story	Children
Escobedo et al [46], 2012	MOSOCO^c^	Smartphones	Communication skills, gaze or eye contact	Phones detect cues in real-life social situations, give feedback for social missteps, and offer advice	Age 8-11 y
Lee [60], 2020	Kinect training system	Video	Social greeting behavior	Trainer “plays” virtual characters in different social interactions with the user to teach appropriate social greeting responses	Age 7-9 y
Lee et al [57], 2018	AR-RPG^d^	Tabletop	Social greeting behavior	Users role-play different social events and different greeting scenarios	Age 7-9 y
Lee et al [61], 2018	ARCM^e^ training system	Tabletop and screen	Social greeting behavior	The user plays roles of different avatars in social situations and needs to choose correct greeting behaviors	Age 8-9 y
Li et al [55], 2021	FaceMe	Touchscreen	Facial or emotional expressions	Minigames, where the user has to remember, choose, and make facial expressions	—^f^
Liu, Salisbury [54], 2017	BPS^g^; FaceGame and Emotion Game	Smart glasses	Facial or emotional expressions, gaze or eye contact	Users learn to gaze at another person and identify emotions of another person’s detected face	Age 8-9 y
Vahabzadeh et al [59], 2018	Empowered Brain or Face2Face module	Smart glasses	Communication skills, gaze or eye contact	Users get prosocial cues through smart glasses during interactions with a trainer, social performance can be seen in digital web portal	Age 6-8 y

^a^ARSFM: augmented reality-based self-facial modeling.

^b^ARVMS: augmented reality-based video modeling storybook.

^c^MOSOCO: mobile social compass.

^d^AR-RPG: augmented reality-tabletop role-playing game.

^e^ARCM: augmented reality concept mapping.

^f^Not available.

^g^BPS: Brain Power System.

Other applications aim to teach gaze direction, such as engaging in mutual eye contact during interactions and being attentive. In *Empowered Brain* [59], the user wears smart glasses while interacting with a facilitator who provides feedback on their head positioning relative to the facilitator and rewards attentive gaze. Similarly, in *BPS* [54], the user is rewarded with points to look at their virtual interaction partners and even receives higher rewards for looking at the socially salient central region of the face. In total, 3 records describe applications for teaching social greeting behavior [57,60,61], in which children learn to identify and use 6 different greeting behaviors (such as nodding their head, shaking hands, or hugging) in role-play scenarios or with virtual characters. Finally, *mobile social compass (MOSOCO)* [46] is the most versatile of the found applications: in this mobile app, 6 basic social skills are trained in a real-world environment by pairing children with autism with interaction partners (without a diagnosis of ASD). The system then detects, for example, eye contact to provide feedback and hints on how to behave in this situation. Other targeted skills include spatial boundaries, conversation initiators, shared interests, appropriate disengagement, and identifying potential communication partners.

### Characteristics of AR Applications to Improve Social Skills in General Populations

We found 7 records describing AR applications in the general population (Table 5). Applications for the general populations are more diverse in terms of outcome goals and target a wider age range than applications for autistic populations. In total, 3 applications were designed for primary school children, whereas 4 targeted an adult population.

**Table 5:** Characteristics of augmented reality application for a general population (n=7).

Study, year	Name	Type	Targeted social skill	Description of application	Target group
Bai et al [47], 2015	FingAR Puppet System	Tabletop	Theory of mind, emotion expression, and understanding	Users use puppets (markers) and a magic wand to see or change the puppets’ emotional expressions on a screen in social pretense play scenarios	Age 4-6 y
Kyungwon et al [45], 2014	AR Petite Theater	Virtual book or smart glasses	Empathy and perspective taking	Users read a story and author the story via changing emotional expressions and dialog, which can also be watched in an animated version	Age 7 y
Lin et al [58], 2020	Empathics System	Smart glasses	Emotion recognition	Smart glasses detect and feed back emotions of conversation partner in real time and visualize emotions for postsessions	Adults
Lorusso et al [49], 2016	Giok	Mobile game	Positive social interactions	Users steer the behaviors and social strategies of Giok the alien to achieve positive outcomes in various social situations	Age 4-5 y
Silva et al [50], 2017	Schizophrenia Simulator	Smart glasses	Stigma reduction and perspective taking	Users experience typical psychotic symptoms of schizophrenia	Students
Swearingen et al [56], 2021	The Woods	Mobile game	Positive social interactions	Collaborative game teaching about social isolation and the importance of social connectedness	Age 8-78 y
Metsiritrakul et al [53], 2016	UP2U	Screen	Gaze and phubbing awareness	Generated avatars show gaze direction of present persons and system teaches about phubbing behavior if people look down instead of to each other	Age 18-58 y

Applications for children target preschool and first-year graders (age 4-7 y). In *AR Petite Theater* [45], children read a story and can then choose suitable emotions for the characters and role-play these emotions, which makes them train their empathic behavior. Similarly, in *FingAR Puppet System* [47], children use a wand to change puppets’ expression while they see themselves and the puppets in a magic mirror. Finally, in *Giok* [49], children followed a fictional character and decided their behavior through multiple-choice questions to lead it through social situations.

*UP2U* [53] describes an application that teaches people about phubbing behavior (ie, focusing on a mobile phone instead of people in a social situation) in public places. The system recognizes the present people and creates an avatar on a screen. Through head tracking, people receive feedback when their heads are not turned toward each other (screen goes red), and a video is played that educates them about phubbing behavior. Lin et al [58] described *Empathics System*, smart glasses that can be used during physician-patient communication (by both physicians and patients). The glasses collect real-time facial emotion information regarding the conversation partner and provide feedback to the user. Although this application mainly supports the current communication situation (ie, supporting performance rather than training), we included this paper because of the described postsessions in which the emotions are visualized so that the user can analyze them after the conversation. Participants also mentioned that the tool has the potential to serve as an educational tool for emerging physicians to learn about emotional cues and their effects. In their *Schizophrenia Simulator*, Silva et al [50] used smart glasses and an immersive AR environment to give users without schizophrenic symptoms an impression of what schizophrenia feels and looks like, with the intention of reducing the stigma around schizophrenia. Finally, *The Woods* [56] is a mobile smartphone game played by 2 players. Unlike other applications, it has a more holistic approach to teaching the importance of positive human connections. Players need to choreograph their movements to interact with virtual objects while engaging in a story about social isolation and reconciliation.

### AR Applications to Improve Social Skills in Both Autistic and General Populations

Of the included records 2 describe applications involving both the autistic and general populations. First, *Empathic Systems* could be used by both patients diagnosed with ASD and physicians. The authors described that participants with autism had many concerns with smart glasses and concluded that their system needs to undergo some adaptations for an autistic population but shows potential in supporting real-life interactions by exemplifying emotions. Second, in *MOSOCO*, children with autism are paired with potential interaction partners without an ASD diagnosis. Although the general population was not the main target group of the study, the authors found that the application supported children without a diagnosis of ASD by destigmatizing missteps of children with autism and reacting in adaptive ways, which led to less teasing.

### Types of AR Used in the Included Applications

The main development software used for the development of the applications was Unity or Vuforia. The included applications used a variety of AR types. Although *FaceMe* and *Giok* use marker-based AR to control virtual expressions and activate gameplay, respectively, and *The Woods* uses projection-based AR to guide players in the room, most of the included applications rely on superimposition AR. They overlay physical objects, other people interacting with the user, or the users themselves with an augmented view.

In the case of physical objects, AR overlays real-world physical scenes, such as books (*AR Petite Theater* and *augmented reality-based video modeling storybook*) or board games (*AR-RPG* and *ARCM*), with virtual scenes that either highlight specific parts or are used to manipulate the real scene. In *augmented reality-based self-facial modeling* and *FingAR* *Puppet System*, users see themselves in a virtual scene to change their facial expressions and see themselves in an AR environment. *UP2U* uses computer vision to create avatars of a group of people that are then displayed on a screen. Finally, some applications use AR in their environment and interaction partners. In *Kinect training system*, the environment is augmented with the required visual background, and in *Schizophrenia Simulator*, psychotic symptoms are reproduced with AR. In *BPS*, human faces are overlaid with an AR cartoon face to engage the user, and *Empowered Brain* uses AR to keep the user attentive to the conversation partner. In *MOSOCO*, AR detects partners and provides support during real-life social situations, and in *Empathics System*, smart glasses detect the partner’s emotions.

### Social Setting of Included AR Applications

Considering the topic of social skills, we also examined the social settings of the applications. Of the 17 included applications, 10 were used as a single-player experience. In addition, 6 applications are performed in dyads, including both interactions with a human facilitator, therapist, or teacher who knows the system and experiences for pairs of naive users (eg, 2 children use the application together [*FingAR Puppet System*] or cooperative gameplay [*The Woods*]). Finally, 1 application (*UP2U*) uses a group setting by analyzing and teaching people to be more attentive to each other.

### Methodological Characteristics of Evaluations of the AR Applications

A summary of the methodological characteristics of the evaluated studies is presented in Table 6. All the included applications have finished prototype development, and only 1 record [51] did not report any kind of evaluation. Most records report some form of co-design for their applications. Applications for autistic populations often include interviews with parents or teachers of the included children. In general, the quality of the evaluation studies was rather low. The paper length varied considerably (between 3 pages and 20 pages, mean 9.5, SD 5.5). Often, authors described the study as “prestudy,” “pilot tests,” or “preliminary tests.” Only 2 studies had a sample size larger than 20 (with 21 and 25 participants). The level of detail reported for how the applications were evaluated varied considerably, including references that stated that it has been tested with users [56] to fully describe the methodology.

**Table 6:** Methodological characteristics of included augmented reality applications (N=17).

Development and study characteristics		Values, n (%)	Reference
**Development and study characteristics**			
	Co-design	6 (35)	[50,52,55,57,58,60]
	Only development	2 (12)	[51,56]
	Usability	12 (71)	[45-47,49,50,53-56,58-60]
	Intended learning outcome	13 (76)	[45-48,50,53-55,57-61]
**Study design**			
	Observation, qualitative, or postquestionnaire	7 (41)	[47,49,53-56,58]
	Pre-post	7 (41)	[46,48,50,57,59-61]
	Pre-post with control group	1 (6)	[45]
**Study sample size**			
	<5	7 (41)	[46,48,54,57,59-61]
	5-10	3 (18)	[52,55,58]
	11-30	5 (29)	[45,47,49,50,53]
	Not specified	1 (6)	[56]
**Study population age (years)**			
	0-6 (early childhood)	3 (18)	[47,49,55]
	7-10 (primary school)	7 (41)	[45,46,54,57,59-61]
	11-14 (early adolescence)	2 (12)	[48,52]
	15-18 (late adolescence)	—^a^	—
	>18 (adult)	2^b^ (12)	[53,58]
	Other	1 (6)	[56]^c^
	Not specified	1 (6)	[50]

^a^Not available.

^b^Students and physicians.

^c^Age 8-78 years.

Both usability and intended outcomes were generally positive in all the studies that tested the applications. A total of 12 records reported usability outcomes. We describe usability as a term that includes usability, feasibility, and acceptability. Moreover, 2 records report that there were no adverse effects and no conflicts; 4 records report that the users were able to understand and complete the intervention (especially in autistic populations), and 8 of the records report that participants found the application interesting and engaging or that users rated the application highly (“excellent” or “brilliant”). Only 1 record (*UP2U*) described the feasibility of the technical side of the application.

A total of 13 records described some form of testing for the intended outcomes. All of them reported positive results concerning their expected outcomes, for example, an increase in social behavior, a decrease in (ASD) symptoms (related to social skills), or verbal affirmation by participants that the application promotes and helps with the intended outcome.

### Additional Findings

Although one of our eligibility criteria was that the application had to have an intended and transferable effect on social skills, during our review process, we found a variety of records that show how AR can be used as support in situations that require social skills or skills related to social skills. As our aim with this scoping review was to give an overview of existing literature, in this section, we want to give some examples of applications supporting users in social situations.

For instance, AR has been found to improve narrative skills during storytelling compared with non-AR story creation, possibly because of the engaging nature of AR [62]. In addition, AR can be used to track the emotions of an audience during a public speaking scenario to reduce fear of speaking by providing direct feedback to the speaker [63]. Furthermore, there is a thin line between improving social skills and just playing a collaborative game. One could argue that just by collaborating, social skills are endorsed and relationships are supported. Some games, for example, *BloxAR* [64], a social game in which teams compete to build AR block structures, use this concept. As we had to balance resources and breadth of inclusion, for this review, we focused on applications that focused on improving social skills in a transferable manner. Further reviews should adopt broader eligibility criteria.

## Discussion

### Overview

In this systematic scoping review, we mapped existing literature on AR applications that improve social skills. We found 17 records that fit our inclusion criteria, that is, reporting AR applications intended to improve social skills as the main goal. As expected due to existing reviews, most AR applications that teach social skills were developed for an autistic population (10/17, 59%). These applications aim to improve emotional expression recognition, social greeting behavior, or gaze direction in children. Applications for the general populations are more diverse and include emotional expression recognition, perspective taking, and empathy, as well as more specific topics such as destigmatization or antiphubbing behavior for both children and adults.

### Principal Findings and Comparison With Prior Work

Most of the records we reviewed focused on developing applications for children with autism, which is reasonable, given that social skills deficits are 1 of the 2 defining characteristics of autism [65]. Our findings are consistent with those of previous reviews, indicating that numerous approaches are being developed for this population. However, we did not find any applications that target adults, suggesting a potential area for future research. When comparing AR applications for autistic populations and general populations, this review showed that facial expressions or emotion recognition and gaze behavior can be found as an outcome goal for both autistic and general populations. Although the application targeting phubbing behavior phrases the outcomes differently, it teaches gaze behavior similar to applications for populations with autism. Applications targeting children are similar for autistic and general populations in terms of intended learning goals and ways to teach these goals. For example, emotional expressions are the main topic addressed in applications for all populations, and allowing children to choose between different emotions is a popular way to teach these expressions.

### The Use of AR in Social Skills Interventions

AR is considered a promising tool for teaching because of its potential for interaction between the virtual and real worlds and its ability to facilitate visualization [66]. The results from our scoping review show that AR for social skills applications is used in 2 different ways: first, as an indispensable part of the application that is crucial for its effects, such as smart glasses that scan the environment and other’s faces, and second, as an additional tool to emphasize important information, animate the scenarios, and generally make the experience more exciting for the user. In these cases, the application does not necessarily rely on AR. Both approaches are valuable, and the positive results concerning usability and intended learning outcomes indicate that AR has a potentially positive effect on learning. However, because most of our included records did not evaluate their applications with a control group, it is not certain whether AR is essential for the intended outcomes. One of our included records [45] compared the AR condition with a non-AR condition for their application and found a significant difference in perspective taking, with children in the AR condition showing higher scores in perspective taking, as well as more engagement. Their sample consisted of 12 children, which shows the need for replications with larger samples.

### Strengths and Limitations of the Included Records

Strengths lie in the inclusion of the target group in the design and development process, the iterative process that many records describe, and the creative detail and hard work that often goes into the development of a serious game or intervention. Limitations are mostly found in the methodology of evaluation of the applications, especially in the small sample size of studies targeting an autistic population, where fewer than 5 children were tested. Evaluations rarely follow rigorous methodology (such as randomized controlled trials). Although iterative development for technical applications is necessary and reporting of preliminary results and evaluations is desirable, our results suggest that systematic and methodologically rigorous evaluations of AR applications for promoting social skills are still lacking.

### Strengths and Limitations of This Scoping Review

This review was conducted in a structured and transparent manner, and the authors tried to eliminate mistakes by repeating screening at the abstract level. The main limitation of this scoping review is that due to the broad field of social skills, our search criteria might have missed some AR applications related to social skills. For example, emotion regulation and empathy are vital skills for adaptive interpersonal relations and should thus be included in the field of social skills. However, owing to the large number of additional records, we did not include these strings as separate search strings. Although being a limitation, some facts show that our search strategy yielded results that appropriately reflect the social skills training field. First, our final data set contains studies related to empathy, which shows that our search had the potential to also find records with this specific skill. In addition, all but one of the records identified through our additional search of existing reviews, reference lists, and follow-up studies were duplicates or had to be excluded, indicating that our search terms were adequate. Further reviews could broaden the search and include specific terms such as “emotional learning” or “empathy.” Second, the interdisciplinarity of the topic and consequent limitations in the databases and search terms used might have resulted in the overlooking of certain records. Finally, the heterogeneity of the included records posed challenges in the synthesizing of the findings. However, different aspects revealed the focus of the current literature, for example, the emphasis on children with autism, which can be valuable for guiding future research.

### Future Directions

Our findings suggest that AR applications have the potential to enhance social skills, as indicated by the evaluations of player experience and usability. Although the intended outcomes were only preliminarily tested in most of the included studies, AR applications can be highly engaging tools for social-emotional learning in psychological, digital, and educational settings.

The search strings “cooperation” and “collaboration” were included because of their common appearance as keywords in AR contexts. The results showed that these 2 strings were not very relevant for our final included records. Social skills are mostly taught in a single-user setting or with a human facilitator. Considering the advantage of AR in easily enabling cooperative or collaborative situations, future research could explore ways to improve social skills in pairs or even groups. The times have passed when the digital world was not social. Nowadays, most digital tools, such as apps and online gaming, include or are exclusively for connecting with other people. However, many of these web-based interactions happen while both or all people are at different locations. AR not only has the advantage of interacting with digital components in the real world but also has the potential for the user to interact with other people while using a digital tool in real life. AR offers the exciting possibility of playing a digital game or interacting with a digital tool while being in the same room with other people who the player interacts with, as such, aiming to improve skills in complex and real-time social situations. As an example, one of our included records, *MOSOCO*, uses AR to teach social interaction skills to children with autism during real-time conversations with other children. Their results show that the app helps both children with autism and neurotypical children during their social interactions with each other. Although the sample size in their study was small, this shows the great potential of such applications to reduce stigma and lead to better social connections in real-world and real-time complex social settings in autistic, general, and mixed populations.

### Conclusions

This scoping review describes the range and characteristics of applications using AR to improve social skills. Applications have been developed for autistic and general populations. Most often, they target emotion recognition, although this varies. The evaluations show promising results in terms of both the usability and intended outcomes of these applications. In future work, more focus should be placed on high-quality evaluations to further substantiate these results, and more complex social interactions in real-world settings should be explored.

## Appendix

Multimedia Appendix 1 PRISMA-ScR (Preferred Reporting Items for Systematic Reviews and Meta-Analyses extension for Scoping Reviews) checklist.

## Abbreviations

AR: augmented reality
ASD: autism spectrum disorder
BPS: Brain Power System
MOSOCO: mobile social compass
PRISMA-ScR: Preferred Reporting Items for Systematic Reviews and Meta-Analyses extension for Scoping Reviews
VR: virtual reality

## Data Availability

The data sets generated and analyzed during this study are available from the corresponding author upon reasonable request.
